# Decision making and executive functions in problematic pornography use

**DOI:** 10.3389/fpsyt.2023.1191297

**Published:** 2023-07-26

**Authors:** Silke M. Müller, Stephanie Antons

**Affiliations:** ^1^Department of General Psychology, Cognition and Center for Behavioral Addiction Research (CeBAR), University of Duisburg-Essen, Duisburg, Germany; ^2^Erwin L. Hahn Institute for Magnetic Resonance Imaging, Essen, Germany

**Keywords:** pornography use disorder, sex addiction, cybersex, problematic usage of the internet, cognition, risky choice

## Abstract

**Introduction:**

Previous research on cognitive functions in Compulsive Sexual Behavior Disorder (CSBD) and problematic pornography use (PPU) reported inconsistent findings and mostly included sexual pictures in the used tasks. The role of general executive functions and cognitive competences (without the presence of appetitive stimuli) in the context of PPU is largely unexplored.

**Methods:**

This study investigated differences between individuals with high versus low problem severity of PPU regarding decision making and executive functions. The sample of the laboratory study consisted of N = 102 male adults. Besides measures of trait impulsivity, we used standard neuropsychological tests (Trail Making Test and three-back working memory task) and an intertemporal risky choice paradigm, the Cards & Lottery Task (CLT).

**Results:**

The results show heightened impulsivity (urgency and deficits in perseverance) in individuals with high PPU, but no differences regarding performance in the CLT and executive function tasks.

**Discussion:**

The findings indicate that PPU might be associated with impulsive behavior when experiencing strong emotions (urgency) and deficient cognitive performance especially in the presence of sexual stimuli but not in general.

## 1. Introduction

Behavioral addictions raise significant public health concerns (1). Research suggests common mechanisms underlying substance-use disorders, such as alcohol use disorder, and non-substance related addictive or maladaptive behaviors, such as gambling disorder (2) or problematic pornography use [PPU; Love et al. (3), Gola et al. (4), and Leeman et al. (5)]. PPU can be considered a subtype of Compulsive Sexual Behavior Disorder (CSBD), which constitutes a large part of the behavioral patterns (6, 7). A growing number of studies support the assumption that PPU may constitute a disorder due to addictive behaviors (8) comparable to the classifications of gambling disorder and gaming disorder in the ICD-11 (9). Accordingly, main features of PPU are considered similar to those of gaming disorder including impaired control over the use, growing priority given to the use, and continuation of use despite the experience of negative consequences. As argued recently, there appears to be enough empirical evidence and theoretical background to consider PPU as a candidate for the ICD-11’s residual category of “other specified disorders due to addictive behaviors” (8).

From a neurocognitive dual-process perspective [e.g., Bechara (10)], addictive behaviors result from an inability to resist temptations, which is characterized by impulsive processes (associated with craving and reward orientation) undermining the control attempts by reflective processes (associated with self-control and executive functions). In other words, an imbalance between (affective) driving paths towards addiction and their (cognitive) counterpart (i.e., self-control) is made responsible for the development of (online) behavioral addictions (11). According to the I-PACE model (12), which is inspired by dual-process approaches, individual predisposing factors, such as trait impulsivity may affect the way in which situational behavior-related cues are perceived. For example, pictures showing sexual content might elicit higher cravings in highly impulsive compared to less impulsive individuals. Cognitive control functions are assumed to moderate the effect of specific cue-induced responses on the actual behavior/ usage. Accordingly, reduced cognitive control abilities are a risk factor for the development and maintenance of addictive behaviors. Executive functions comprise a diversity of cognitive control processes with varying complexity (13, 14). These “cold” functions include monitoring and rapid change of content in working memory as well as flexibly switching between tasks or mental sets and play a key role in decision making as they enable to deliberate about options and adapt behavior [see also (15–17)]. Meta-analytical results by Ioannidis et al. (18) show that individuals with (unspecified) problematic Internet use show deficits in executive functions (working memory, cognitive flexibility, motor inhibition) as well as deficits in risky decision making measured by tasks such as the Iowa Gambling Task (IGT), Balloon Analogue Risk Task (BART), Cambridge Gambling Task (CGT), and Game of Dice Task (GDT) – Tasks for which reduced performance has also been reported in individuals with substance use disorders (19).

In the context of PPU, research on decision making and executive functions is relatively scarce. Castro-Calvo and colleagues (20) reviewed experimental studies and summarized that PPU is associated with attentional bias towards sexual cues as well as with reductions in executive functions (especially motor inhibition and working memory) and decision making. Looking more closely, most of the studies used tasks that included sexual stimuli. For example, the study by Sinke et al. (21) indicates that men with PPU, as compared to healthy controls, only showed deficits in a working memory task when sexual pictures were presented in the background, and not when neutral pictures were presented. Similar interferences were shown for other executive function tasks [e.g., Schiebener et al. (22) and Wang and Dai (23)] and decision making tasks [IGT with pornographic and neutral cues; Laier et al. (24)]. Other PPU studies that the review summarized under “decision making” used either Approach/Avoidance tasks (25–28) or delay discounting tasks (29, 30). The former represents implicit behavioral tendencies while the latter represents a measure of trait impulsivity (the tendency to prefer smaller sooner over larger later rewards), which both is to be distinguished from risky choice. The only study mentioned using a risky decision-making task (IGT) without interference of sexual stimuli (31) compared hypersexual with control participants (all male). The groups did not differ with regard to decision-making performance (i.e., number of choices for advantageous versus disadvantageous decks; groups only differed with regard to gain/loss frequency). Looking more broadly at (further) studies of problematic/ compulsive sexual behavior draws an inconsistent picture. Some studies reported reduced executive functions (working memory, cognitive flexibility, and motor inhibition) and poorer decision-making performance (CGT) in individuals with CSBD compared to control participants (31–33). Contrarily, others reported no such differences using very similar measures (34, 35). What virtually all these studies report consistently is that PPU is associated with heightened trait impulsivity [see also (36, 37)]. Heightened impulsivity also characterizes problematic usage of the Internet (38) and a variety of other psychiatric disorders.

Taken together, reductions in executive functions and risky decision-making, and elevated impulsivity appear to be critically involved in addictive behaviors. Research on the impact of these functions in PPU is scarce and results are mixed. This study investigates risky decision making and executive functions in PPU. To take the intertemporal nature of decision-making in addiction into account (i.e., preferring short-term gratification by neglecting long-term risks), we used a risky decision-making task including conflicting short- and long-term consequences: the Cards and Lottery Task (CLT). The CLT represents decision making in situations, in which striving for immediate gratification increases the risk of negative long-term outcomes, while resisting immediate temptations increases the likelihood of positive long-term outcome. We hypothesized that individuals with PPU show deficits in the CLT as compared to individuals without PPU. We further expected PPU to be associated with deficits in other cognitive domains as well as with increased trait impulsivity.

## 2. Methods

### 2.1. Participants

The study was part of a larger study series that consisted of three parts: (1) an online survey, (2) an experimental study focusing on the relevance of (a) stress and (b) executive functions in PPU, and (3) an fMRI study. Each study part had individual research questions. Results from the online survey (36, 39), the stress part of the experimental study (40) and the fMRI study (41) have been published previously. Participation in the respective next part was optional. The study was conducted 2017 to 2018 and focused on heterosexual male participants since explicit pornographic material tailored for this target group was shown in some tasks. Recruitment was carried out via newsletters and nationwide media articles. The study parts had related but individual research questions. Results from (1) the online survey and (3) the fMRI study parts have been published previously (36, 39, 41).

Overall, 1,498 participants finalized the online survey. All participants were asked if they are interested in participating in the experimental study. Those who were interested, were contacted, informed about the study, and invited to the laboratory. Overall, 107 participated in the laboratory study. For *n* = 5, data was incomplete due to technical problems. The final sample of the laboratory part consisted of 102 participants (*M*_age_ = 29.14, *SD*_age_ = 9.22, range_age_: 18–62; all male). Most participants described themselves as clearly heterosexual (93.1%) while the rest indicated a more heterosexual orientation (6.9%). Overall, participants had a high education with 75.5% having a high school degree. Over half of the participants (51%) indicated to be in a romantic relationship at the time of study participation. The short Internet Addiction Test modified for pornography use [sIATporn; Laier et al. (42)] was used to identify extreme groups, namely a high problem severity group (sIATporn>29, *n* = 34) and low problem severity group (sIATporn<21, *n* = 36).

### 2.2. Procedure

**Figure 1:**
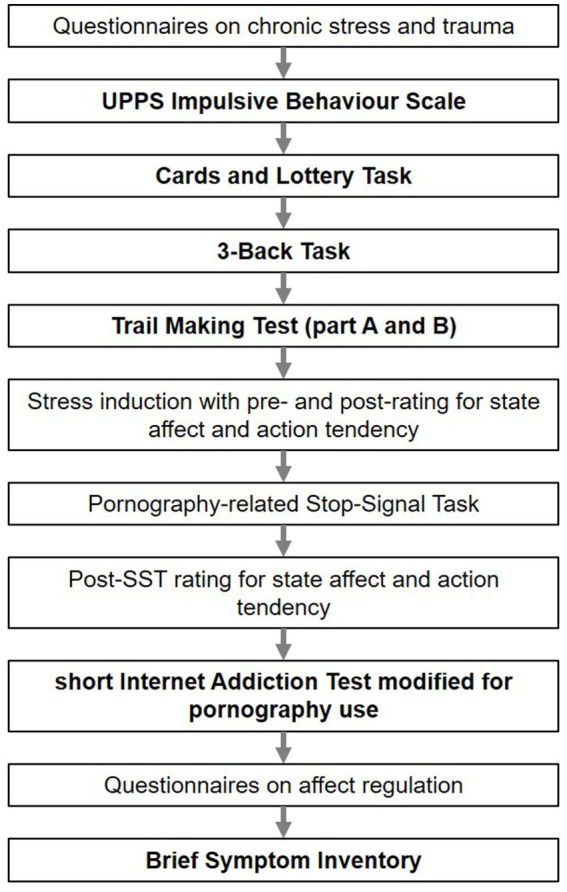
Procedure of the laboratory study. Study parts relevant for the current analyses are written in bold.

The laboratory study had two aims: besides investigating executive functions in the context of PPU, we also aimed to investigate effects of stress on pornography-related response inhibition. We here focus on the results on executive functions. Executive functions were assessed before the stress induction and should therefore not be biased by the experimental manipulation. Nevertheless, the pre-stress induction ratings on state affect were used as covariates in the analyses. See Figure 1 for an overview on the procedure.

#### 2.2.1. Questionnaires

##### 2.2.1.1. Problem severity of pornography use

The 12-Item short Internet Addiction Test modified for pornography use [sIATporn, Laier et al. (42)] was used to assess a problem severity of pornography use. At the time of the study, the new ICD-11 criteria for CSBD and PPU had not yet been published. The sIATporn assesses the problem severity on two subscales: control/time management and craving/social aspects. The questionnaire includes 12 items answered on a scale from 1 (never) to 5 (very often) resulting in sum scores ranging from 12 to 60. Comparing the sIATporn items with the ICD-11 criteria for CSBD there is an overlap on three criteria: (1) behavior has become central focus of the individual’s life to the point of neglecting health and personal care or other interests, activities and responsibilities (sIATporn item: e.g., “How often do you neglect household chores to spend more time on Internet pornography?”), (2) The individual has made numerous unsuccessful efforts to control or significantly reduce repetitive sexual behavior (sIATporn item: e.g., “How often do you try to cut down the amount of time you spend on Internet pornography and fail?”), and (3) The individual continues to engage in repetitive sexual behavior despite adverse consequences (sIATporn item: e.g., “How often do your grades or school work suffer because of the amount of time you spend on Internet pornography?”). The ICD-11 criteria on continuation even when deriving little or no satisfaction from it and marked distress or significant impairment are not assessed with the sIATporn. For the current analyses, the sum-score was used with higher score indicating higher problem severity. The sum score and subscales of the sIATporn had high internal consistency in the current sample Cronbach’s *α* = 0.897 (control/time management: 0.885; craving/social aspects: 0.801).

##### 2.2.1.2. Impulsivity

The degree of impulsivity was assessed with the German version of the UPPS Impulsive Behavior Scale (43). The questionnaire consists of 45 items answered on a 4-point Likert scale ranging from 1 (strongly agree) to 4 (strongly disagree). Four facets of impulsivity are assessed with the UPPS: urgency (e.g., “When I feel rejected, I will often say things that I later regret.”), lack of premeditation (e.g., “I usually think carefully before doing anything.”), lack of perseverance (e.g., “I generally like to see things through to the end.”), and sensation seeking (e.g., “I quite enjoy taking risks.”). Mean values ranging from 1 to 4 are calculated for each subscale. Higher scores indicate higher degree of impulsivity. The subscales had good to high internal consistency (Cronbach’s α: urgency = 0.881, lack of premeditation = 0.840, lack of perseverance = 0.809, sensation seeking = 0.737) in the current sample.

##### 2.2.1.3. Further variables

To further describe the sample and extreme groups, as well as to identify confounding variables the general degree of psychopathological symptoms was assessed with the Brief Symptom Inventory [BSI; Derogatis and Spencer (44)]. The subscales for anxiety and depression are possible confounding variables, as both have been associated with executive functions and decision making abilities (45) and may also be relevant in PPU (46, 47). As further potential confounding variables, the current degree of anxiety was assessed with the state subscale of the State–Trait-Anxiety Inventory [STAI, state anxiety; Spielberger et al. (48)] and the current degree of urge to use pornography was assessed with a single item “Please indicate your current urge to use pornography” answered on a scale from 0 (=no urge at all) to 100 (=very strong urge).

#### 2.2.2. Experimental tasks

##### 2.2.2.1. Decision making

The Cards and Lottery Task [CLT; Mueller et al. (49) and Schäfer et al. (50)] is a computerized intertemporal risky choice task. In the CLT, each decision has an effect with respect to both a short-term outcome (i.e., immediate gain/loss) and a long-term outcome (i.e., gain/loss of a jackpot at the ed. of the game). Participants are instructed to repeatedly choose from which of two decks of cards a card should be drawn at random. Each deck contains ten cards with different values and symbols. A drawn card effects the game in two ways: (1) the card’s value (positive/ negative) is charged directly to the current account balance leading to an immediate gain/ loss, additionally, (2) the card’s symbol (star/ bomb/ no symbol) effects the probability of gaining/ losing a large amount of virtual money in a final lottery. Symbol cards are collected in an extra account and increase (stars) or decrease (bombs) the chance to win the lottery. In each of the 36 rounds, the two decks are renewed and thus vary in the number and height of possible outcomes. The CLT provides explicit information about the possible gain/ loss amounts and number of star/ bomb cards included in the two decks. However, the value-symbol-constellations do not vary completely randomly: The left deck tends to provide high immediate gains but, at the same time, many bomb cards (i.e., it is unfavourable in the long term) while the right deck tends to offer low immediate gains or even losses but, at the same time, many star cards (i.e., it is favourable in the long term). The CLT with all its contingencies is described in detail by Mueller et al. (49). The main scores represent the number of choices of the right over the left deck (CLT_netscore; possible range: −36 to 36), the expected value of the final outcome (CLT_EV; i.e., final account balance plus expected lottery outcome), and the number of advantageous decisions (CLT_NAD) which can reach values between 0 and 36. The CLT_NAD is different from the CLT_netscore because choosing the right deck is not always advantageous [see (49)].

##### 2.2.2.2. Working-memory performance

The three-back task was used to assess working memory performance. The computerized task consists of 5 blocks with 24 trials each. In each trial, participants are confronted with single digits (0–9) displayed in random order for 500 ms. Participants have to indicate by button press, if the current digit was presented exactly three trials before or not. After the digit presentation a fixation cross was presented for 2.75 ms followed by feedback on whether the response was correct (green check mark) or not (red cross). Working memory performance is indicated by the percentage proportion of correct responses of the last four blocks, with higher scores indicating better working-memory performance.

##### 2.2.2.3. Cognitive flexibility and information processing speed

The Trail Making Test [TMT, parts A and B; Reitan (51)] was used to assess cognitive flexibility and information processing speed. As paper pencil task, participants had to join letters (part A) as well as letters and numbers in an alternating order (part B) with a drawn line. The seconds needed to complete the tasks were used as performance measure, with longer time needed indicating worse performance.

### 2.3. Statistical analyses

Statistical analyses were carried out with IBM SPSS Statistics version 28. For correlational analyses, Pearson’s correlations and confidence intervals (95%) based on bootstrapping were estimated. As described earlier, extreme groups were built based on the upper and lower sIATporn terciles. Group differences regarding descriptive/ predisposing variables were tested using *t*-tests with Bonferroni correction. For hypotheses testing, differences between groups regarding decision making, executive functions, and impulsivity were analyzed with ANCOVA. We accounted for differences in predisposing variables by using those that differed between groups as covariates, namely BSI: Depression, BSI: Anxiety, Urge to use, STAI: State. Effect sizes of group differences are indicated by partial ƞ^2^.

## 3. Results

### 3.1. Descriptive statistics

Descriptive statistics and group differences with regard to psychopathological symptoms, affective state and pornography use pattern are presented in Table 1. Individuals with high problem severity (high sIATporn) show significantly higher levels of depression, anxiety, current urge to use pornography, state anxiety, and duration of pornography use compared to individuals with low problem severity (low sIATporn). Groups did not differ with regard to age and frequency of use. No differences between groups were found with regard to educational level (χ^2^ = 1.12, *p* = 0.773). Descriptive statistics on measures of decision making and executive functions are presented in Table 2.

**Table 1:** Descriptive statistics of the main variables overall and per group of individuals with high and low problem severity of PPU.

Variables					Severity of PPU							
	Overall *n* = 105				High sIATporn *n* = 34		Low sIATporn *n* = 36		Difference tests			
	min	max	*M*	SD	*M*	SD	*M*	SD	df	*t*	*p*	*d*
Age	18.00	62.00	29.13	9.22	29.68	7.83	27.11	9.74	68.00	1.21	0.115	0.29
sIATporn	13.00	55.00	26.68	8.84	37.00	5.77	17.92	2.41	46.61^a^	17.86	<0.001	4.36
BSI: depression	0.00	18.67	4.56	4.55	7.14	5.37	3.11	3.10	52.20^a^	3.81	<0.001	0.93
BSI: anxiety	0.00	12.33	3.43	2.94	4.61	3.02	2.45	2.24	68.00	3.42	0.001	0.82
Urge to use	0.00	86.00	20.09	22.41	31.50	26.31	8.53	9.06	40.33^a^	4.83	<0.001	1.18
STAI: state	20.00	68.00	40.50	9.18	45.15	9.38	37.64	8.71	68.00	3.47	<0.001	0.83
Frequency of use	0.00	500.00	11.65	49.27	23.32	84.40	4.21	3.88	68.00	1.36	0.179	0.33
Duration per use	2.00	150.00	35.05	28.77	49.62	36.56	25.56	13.62	41.54^a^	3.61	<0.001	0.88

PPU, pornography use disorder; sIATporn, short internet addiction test specified for pornography use; BSI, brief symptom inventory; STAI, state–trait anxiety inventory.

a Corrected degrees of freedom due to differences in variances between groups as indicted by Levene’s test for equality of variances.

**Table 2:** Descriptive statistics and group differences for low and high problem severity groups and measures of executive functions.

	Overall				Severity of PPU				Group differences			Significant covariates
					High sIATporn		Low sIATporn					
	min	max	*M*	SD	*M*	SD	*M*	SD	*F*	*p*	Part. ƞ^2^	
CLT_NAD	6.00	31.00	21.43	6.70	21.68	5.64	21.58	7.00	0.95	0.334	0.015	
CLT_netscore	−36.00	36.00	5.15	15.85	5.74	14.59	5.67	15.95	0.55	0.460	0.009	
CLT_EV score	−1571.00	8094.00	3135.83	2233.00	3428.50	2018.14	2888.75	2417.69	0.14	0.709	0.002	
Three back: % correct	0.00	0.98	0.62	0.18	0.62	0.17	0.67	0.17	0.35	0.555	0.006	
TMT_A: time in sec.	11.18	74.00	24.74	9.21	24.92	7.66	25.25	12.14	1.38	0.245	0.021	
TMT_B: time in sec.	18.74	128.27	51.31	18.43	52.65	17.54	48.64	17.13	0.139	0.710	0.002	
UPPS: urgency	1.25	3.50	2.49	0.54	2.83	0.46	2.25	0.47	6.97	0.010	0.100	Age, BSI: depression
UPPS: deficits in premeditation	1.00	3.18	2.00	0.47	2.04	0.47	2.04	0.43	1.56	0.286	0.018	
UPPS: deficits in perseverance	1.00	3.20	2.09	0.47	2.26	0.45	1.94	0.46	2.40	0.126	0.037	STAI: state
UPPS: sensation seeking	1.33	3.83	2.80	0.49	2.83	0.42	2.87	0.50	0.37	0.543	0.006	

CLT, cards & lottery task; NAD, number of advantageous decisions; EV, expected value; TMT, trail making test; UPPS, urgency, (lack of) premeditation, (lack of) perseverance, and sensation seeking impulsive behavior scale.

### 3.2. Differences in decision making, executive functions, and impulsivity

As shown in Table 2, individuals with high problem severity do not differ from those with low problem severity regarding performance in the decision-making and executive function tasks (CLT, three-back, TMT). With regard to impulsivity, significant differences could be identified in the UPPS’s subscales urgency, with the high problem severity group scoring higher than the low problem severity group (Table 2). The covariates age and BSI-depression were significant for this comparison. Regarding perseverance impulsivity, effects did not reach significance when correcting for covariates, STAI: State could be identified as significant covariate, but a significant effect was found when using a simple *T*-test without covariates (*t* (68) = 3.00, *p* = 0.004, Cohen’s *d* = 0.72).

### 3.3. Exploratory correlation analysis

Table 3 shows correlations between problem severity of PPU and variables describing the excessive engagement in pornography use (frequency of use, duration per use) with measures of decision making, executive functions and impulsivity. Problem severity significantly correlated with UPPS urgency and deficits in perseverance. No correlations between problem severity and measures of decision making and executive functioning could be identified. Interestingly, frequency of pornography use was significantly and negatively associated with the CLT_netscore indicating that less frequent pornography use is associated with the tendency to resist immediate temptations in order to achieve positive long-term outcomes (higher CLT_netscore). Furthermore, use frequency was positively associated with deficits in perseverance impulsivity with small to moderate effect sizes (see Table 3).

**Table 3:** Correlations between problem severity and use patterns with measures of executive functions.

		1	2	3	4	5	6	7	8	9	10	11	12
1	sIATporn	–											
2	Frequency of use	0.204*	–										
		[0.170, 0.513]											
3	Duration per use	0.331**	−0.075	–									
		[0.176, 0.478]	[−0.154, 0.270]										
4	CLT_NAD	0.001	−0.189	−0.087	–								
		[−0.199, 0.183]	[−0.365, 0.173]	[−0.294, 0.139]									
5	CLT_netscore	−0.020	−0.211*	−0.111	0.972**	–							
		[−0.215, 0.165]	[−0.401, 0.159]	[−0.342, 0.133]	[0.957, 0.985]								
6	CLT_EVScore	0.065	−0.182	−0.117	0.785**	0.741**	–						
		[−0.155, 0.281]	[−0.346, 0.149]	[−0.327, 0.104]	[0.682, 0.857]	[0.616, 0.829]							
7	Three back: % correct	−0.116	−0.063	0.051	0.190	0.164	0.148	–					
		[−0.296, 0.070]	[−0.228, −0.011]	[−0.108, 0.210]	[0.009, 0.369]	[−0.027, 0.351]	[−0.047, 0.342]						
8	TMT_A	0.053	0.094	−0.063	0.037	0.007	−0.070	−0.072	–				
		[−0.114, 0.258]	[−0.117, 0.233]	[−0.200, 0.103]	[−0.178, 0.199]	[−0.196, 0.165]	[−0.231, 0.081]	[−0.236, 0.071]					
9	TMT_B	0.085	0.047	0.165	−0.191	−0.216*	−0.232*	−0.382**	0.384**	–			
		[−0.102, 0.276]	[−0.088, 0.234]	[−0.064, 0.401]	[−0.402, 0.049]	[−0.436, 0.053]	[−0.425, −0.014]	[−0.548, −0.196]	[0.089, 0.608]				
10	UPPS: urgency	0.487**	0.004	0.052	0.047	0.076	0.036	−0.136	0.106	<0.001	–		
		[0.320, 0.627]	[−0.047, 0.393]	[−0.123, 0.217]	[−0.134, 0.207]	[−0.103, 0.235]	[−0.164, 0.216]	[−0.316, 0.069]	[−0.046, 0.279]	[−0.177, 0.188]			
11	UPPS: deficits in premeditation	0.010	0.169*	−0.038	−0.008	0.035	−0.053	−0.143	−0.062	0.027	0.264**	–	
		[−0.188, 0.224]	[−0.117, 0.349]	[−0.162, 0.207]	[−0.188, 0.178]	[−0.140, 0.219]	[−0.233, 0.140]	[−0.338, 0.060]	[−0.206, 0.099]	[−0.134, 0.194]	[0.077, 0.447]		
12	UPPS: deficits in perseverance	0.277**	0.016	0.021	0.158	0.187	0.197*	−0.002	0.009	−0.033	0.449**	0.209*	–
		[0.114, 0.431]	[−0.024, 0.219]	[−0.162, 0.207]	[−0.022, 0.332]	[0.005, 0.358]	[0.023, 0.373]	[−0.162, 0.168]	[−0.130, 0.174]	[−0.203, 0.151]	[0.251, 0.611]	[0.033, 0.371]	
13	UPPS: sensation seeking	−0.046	−0.057	−0.061	0.149	0.161	0.152	−0.184	−0.111	0.052	−0.073	0.282**	0.282**
		[−0.241, 0.148]	[−0.145, 0.321]	[−0.216, 0.108]	[−0.056, 0.324]	[−0.035, 0.330]	[0.092, −0.045]	[−0.369, 0.027]	[−0.275, 0.101]	[−0.120, 0.232]	[−0.261, 0.122]	[0.087, 0.455]	[0.087, 0.455]

The 95% confidence intervals of Bootstrap procedure are presented in squared brackets. CLT, cards & lottery task; NAD, number of advantageous decisions; EV, expected value; TMT, trail making test; UPPS, urgency, (lack of) premeditation, (lack of) perseverance, and sensation seeking impulsive behavior scale. *p < 0.01, **p < 0.001.

## 4. Discussion

This study investigated executive functions and decision making under objective risk in individuals with high compared to low severity in PPU. The extreme-group comparisons indicate that high PPU severity is associated with heightened psychopathological symptoms (depression and anxiety), heightened facets of trait and state impulsivity and elevated use duration (but not frequency). Contrary to our hypothesis, the groups did not differ regarding performance in measures of executive function and decision-making performance.

The findings are in line with previous studies showing increased impulsivity in PPU [although impulsivity might play a more prominent role in hypersexual behavior than in PPU; Bőthe et al. (52)]. Heightened impulsivity is also characteristic for other specified domains of problematic Internet use, such as gaming disorder (53) or problematic social-network use (54–57) as well as for other types of addictive behaviors like gambling disorder or substance use disorders [for reviews see (2, 58)]. This is consistent with the assumptions of theoretical models of addiction [e.g., Bechara (10), Brand et al. (12), Wiers et al. (59), and Everitt and Robbins (60)]. Neuroscientific research shows functional and structural differences in brain regions associated with the “reward system” in individuals with compared to those without PPU (7, 61).

Individuals with higher severity of PPU, as compared to those with low problem severity, performed equally well in the tasks measuring risky decision-making (CLT) and executive functions (three-back and TMT). Accordingly, individuals with high problem severity for PPU do not appear to have any significant deficits in weighing long-term versus short-term consequences. These results are in contrast to empirical findings on gambling disorder and gaming disorder, which are commonly characterized by impairments in risky decision-making and executive functions (58, 62). This trend is also reflected in the results of a large meta-analysis of problematic online behaviors, which included mainly studies on gaming (18). Previously, PPU has also been associated with deficits in executive functions, including inhibitory control and working memory, and decision making (20), which appears to be contrary to the results of this study. Looking more closely, the great majority of the previous studies on executive functions (e.g., inhibitory control, working memory) in PPU used tasks with pornographic pictures (as stimuli or in the background) indicating deficits in case arousing pictures are present. These findings point at stimuli-specific reductions in cognitive/inhibitory control which, according to the I-PACE model, emerge in later phases of addiction development (12). Part of the participants in this study underwent an additional fMRI part including a modified Stop-Signal-Task, the results of which have been reported previously (41). At the behavioral level, symptom severity was associated with faster reaction times, indicating better (and not reduced) inhibitory control, which might be explained by increased insula activity during inhibitory control processing (41). When individuals were under acute stress a higher symptom severity was associated with longer reaction times, indicating reduced inhibitory control (40). These results indicate that, situational factors might impact executive control abilities. Deficits in decision making have previously mainly been referred to a preference for smaller sooner over larger later rewards (i.e., increased delay discounting), which captures individuals’ trait impulsivity rather than decision-making competence (63), or as a result of the interference between presentation of pornographic pictures and risky choice options [see (20)]. The latter can be assumed to result from biases towards pornography-related stimuli, which divert attention from decision-relevant content leading to reduced performance in such tasks.

In the absence of addiction-related stimuli, however, as it was the case in this study, individuals with PPU do not seem to make more risky/disadvantageous decisions. Similar findings have been reported for other types of problematic Internet use apart from gaming (64–66). In accordance with findings from a recent meta-analysis (67), risky decision-making tendencies might be a specific feature of gaming disorder, and not (or not so markedly) of other internet-related disorders such as problematic social networks use or PPU. Also, our results indicate that general working memory and cognitive flexibility (measured with tasks that do not present pornographic content) might not be impaired in PPU. However, studies on PPU that used cognitive measures without pornographic pictures, are yet very rare. Further research is needed to determine the extent and specificity of cognitive impairment in individuals with PPU.

A limitation of the current study is the non-clinical sample. The instrument used to identify high and low severity of PPU (sIATporn) has been used previously, however, it does not allow valid classification. From today’s point of view, we would recommend instruments that have been developed based on current diagnostic criteria for CSBD [e.g., the Compulsive Sexual Behavior Disorder Scale, CSBD-19; Bőthe et al. (68)] or for disorders due to addictive behaviors [e.g., the Assessment of Criteria for Specific Internet Use Disorders, ACSID-11; Müller et al. (69)]. Furthermore, the sample included only male participants, which limits the generalizability of the current findings. The restriction to exclusively male subjects was due to the design of the additional fMRI examination [described in Antons and Brand (41)]. Although some differences between men and women with CSBD are identified, impulsivity is associated with CSBD and PPU regardless of gender (70). The role of decision making and executive functions in women with PPU needs to be explored in future research.

## Data availability statement

The raw data supporting the conclusions of this article will be made available by the authors, without undue reservation.

## Ethics statement

The studies involving human participants were reviewed and approved by Ethics committee of the Department of Computer Science and Applied Cognitive Science at the University of Duisburg-Essen. The patients/participants provided their written informed consent to participate in this study.

## Author contributions

SA conceptualized and carried out the study and analyzed the data. SM wrote the first draft of the manuscript that was reviewed by SA. All authors contributed to the article and approved the submitted version.

## Funding

The work of SM and SA on this article was carried out in the context of the Research Unit ACSID, FOR2974, funded by the Deutsche Forschungsgemeinschaft (DFG, German Research Foundation) – 411232260.

## Conflict of interest

The authors declare that the research was conducted in the absence of any commercial or financial relationships that could be construed as a potential conflict of interest.

## Publisher’s note

All claims expressed in this article are solely those of the authors and do not necessarily represent those of their affiliated organizations, or those of the publisher, the editors and the reviewers. Any product that may be evaluated in this article, or claim that may be made by its manufacturer, is not guaranteed or endorsed by the publisher.
